# Anti-metastatic effects of viral and non-viral mediated Nk4 delivery to tumours

**DOI:** 10.1186/1479-0556-7-5

**Published:** 2009-03-09

**Authors:** Alexandra Buhles, Sara A Collins, Jan P van Pijkeren, Simon Rajendran, Michelle Miles, Gerald C O'Sullivan, Deirdre M O'Hanlon, Mark Tangney

**Affiliations:** 1 Cork Cancer Research Centre, Mercy University Hospital, Leslie C Quick Junior Laboratory, University College Cork, Cork, Ireland

## Abstract

The most common cause of death of cancer sufferers is through the occurrence of metastases. The metastatic behaviour of tumour cells is regulated by extracellular growth factors such as hepatocyte growth factor (HGF), a ligand for the c-Met receptor tyrosine kinase, and aberrant expression/activation of the c-Met receptor is closely associated with metastatic progression. Nk4 (also known as Interleukin (IL)32b) is a competitive antagonist of the HGF c-Met system and inhibits c-Met signalling and tumour metastasis. Nk4 has an additional anti-angiogenic activity independent of its HGF-antagonist function. Angiogenesis-inhibitory as well as cancer-specific apoptosis inducing effects make the Nk4 sequence an attractive candidate for gene therapy of cancer. This study investigates the inhibition of tumour metasasis by gene therapy mediated production of Nk4 by the primary tumour. Optimal delivery of anti-cancer genes is vital in order to achieve the highest therapeutic responses. Non-viral plasmid delivery methods have the advantage of safety and ease of production, providing immediate transgene expression, albeit short-lived in most tumours. Sustained presence of anti-angiogenic molecules is preferable with anti-angiogenic therapies, and the long-term expression mediated by Adeno-associated Virus (AAV) might represent a more appropriate delivery in this respect. However, the incubation time required by AAV vectors to reach appropriate gene expression levels hampers efficacy in many fast-growing murine tumour models. Here, we describe murine trials assessing the effects of Nk4 on the spontaneously metastatic Lewis Lung Carcinoma (LLC) model when delivered to primary tumour via plasmid lipofection or AAV2 vector. Intratumoural AAV-Nk4 administration produced the highest therapeutic response with significant reduction in both primary tumour growth and incidence of lung metastases. Plasmid-mediated therapy also significantly reduced metastatic growth, but with moderate reduction in primary subcutaneous tumour growth. Overall, this study demonstrates the potential for Nk4 gene therapy of metastatic tumours, when delivered by AAV or non-viral methods.

## Findings

HGF is a heterodimeric molecule and functions include mitogenic, motogenic, morphogenic and anti-apoptotic activities [1,2]. HGF plays roles in organizing tissues during development and regeneration, but in cancer stimulates malignant cell invasive behaviour [3-5]. Nk4 consists of the N-terminus of HGF (447 amino acids of α-chain), which contains an N-terminal hairpin and four kringle domains (β-chain removed) [6]. This molecule inhibits cell proliferation and induces apoptosis by the first kringle domain [7] and promotes anti-angiogenic activities through the competitive inhibition of binding of angiogenic growth factors to endothelial cells by its N-terminus [8].

**Figure 1:**
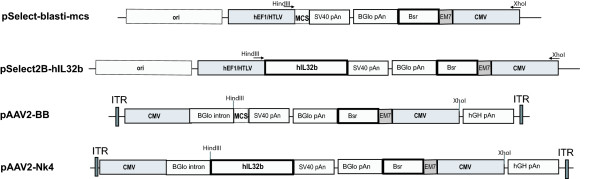
**Vector Constructs**. pSelectBlasti-MCS and pSelectBlasti-2BhIL32b were purchased from Invivogen (Cayla SAS, Toulouse, France). Coding sequences (*IL32 *and *Bsr *= Blasticidin resistance gene) are indicated in dark outline. The functionality of the human IL32b sequence in mice has previously been published [16]. The CMV and hEF1/HTLV composite promoters are indicated in grey. For AAV vector constructs, the *IL32 *(Nk4) expression cassette including the blasticidin resistance gene was PCR amplified using primers designed with a XhoI and HindIII restriction site overhang, (forward-hindIII: 5'AGCAGCAGCTTCCCTGCTTGCTCAACTCTAC3', reverse-xhoI: 5'AGCAGCCTCGAGCAGGCGTTACATAACTTACGG3'and cloned into pAAV-MCS. Clone sequences were validated by sequencing (MWG Biotech).

**Figure 2:**
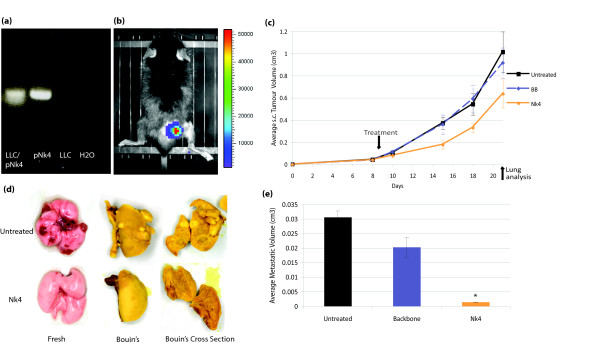
**Plasmid mediated Nk4 gene therapy of LLC tumours**. ***(a) Nk4 plasmid expression in vitro ***LLC cells were transfected with pSelectBlasti-2BhIL32b by lipofection *in vitro*. cDNA was prepared from total RNA extracted at 24 h, and subjected to PCR with primers specific for a 300 bp Nk4 sequence (5'CCTCTCTGATGACATGAAGAAG3', 5'TGTCACAAAAGCTCTCCCC3'). Lane 1 = RNA from LLC transfected with pSelectBlasti-2BhIL32b, lane 2 = pSelectBlasti-2BhIL32b DNA, lane 3 = RNA from untransfected LLC cells, lane 4 = H_2_O template control. **(b) *Transfection of LLC tumours in vivo ****In vivo *luciferase activity from pCMV *Luc *transfected tumours was analysed. 100 μl 6 mg/ml luciferin (Biosynth, Switzerland) was injected i.p. and i.t. Mice were anaesthetised by i.p. administration of 100 μg xylazine and 1 mg ketamine. Ten minutes post-luciferin injection, mice were imaged for 1 min using an intensified CCD camera (IVIS Imaging System, Xenogen). 1.64 × 10^-8 ^p/sec/cm^2^/sr/plasmid copy was observed. **(c) *Tumour growth curve of LLC treated tumours ***Time points of treatment and lung excision are indicated. Nk4 treated group showed reduction in tumour size compared with the other control groups, indicating Nk4 effect on tumour growth although not statistically significant. (n = 6) **(d) *Macroscopic LLC metastatic lung nodules ***Nodules appear as dark red spots on freshly excised lung, or light yellow colour on lungs fixed in Bouin's solution O/N. Cross sections show the morphological appearance of tumours on the inside of the lungs. Lungs were harvested from mice at day 21 post tumour induction. **(e) *Average volume of lung metastases ***The Nk4 group exhibited a significant reduction in metastatic burden compared with control groups (n = 3). Significant difference was observed in the volume of metastases between the Nk4 treated group compared with both the untreated group (p = 0.004), and the backbone group (p = 0.029). No statistical difference was observed between backbone and untreated groups (p = 0.587).

This study describes murine trials assessing the effects of Nk4 gene therapy on the spontaneously metastatic murine LLC model when delivered to the primary tumour via plasmid lipofection or AAV2 vector. DNA constructs are shown in figure 1. The Nk4 expressing plasmid pSelectBlasti-2BhIL32b and the equivalent backbone pSelectBlasti-MCS were purchased from Invivogen (Cayla SAS, Toulouse, France). LLC cell line was purchased from ATCC and maintained according to ATCC recommendations. In order to administer gene as early as possible in tumour growth (smallest injectable tumour size), plasmid DNA (prepared using Endotoxin free Plasmid MegaPrep Kit (Qiagen, West Sussex, UK)) was delivered to tumours using Lipofectamine2000 (Invitrogen Corp., Paisley, Scotland) at day 7-post tumour induction. The transfectability of LLC with lipofectamine 2000 was demonstrated *in vitro *with pEGFP-F delivery as assessed by fluorescent microscopy (data not shown), and *in vivo *(figure 2b). Nk4 expression in pSelectBlasti-2BhIL32b transfected LLC cells was demonstrated *in vitro *by RT-PCR (figure 2a). All *in vivo *experiments were approved by the ethics committee of University College Cork. Subcutaneous (s.c.) LLC tumours were induced in 6–8 week old female C57 mice obtained from Harlan Laboratories (Oxfordshire, England) using 2 × 10^5 ^cells, suspended in 200 μl serum free Dulbecco Modified Eagle Medium, DMEM, (GIBCO, Invitrogen Corp., Paisley, Scotland) injected subcutaneously into the flank. When the tumours reached an average volume of 0.1 cm^3^, they were intratumourally (i.t.) administered 75 μl plasmid/lipofectamine2000 mix containing 25 μg DNA corresponding to Nk4-coding or Backbone (BB) plasmid, or received no treatment (n = 9). The firefly luciferase coding plasmid pCMVluc (Plasmid Factory, Germany) was also administered to a group to validate transfection (n = 3) and IVIS imaged at 24 h. 1.64 × 10^-8 ^p/sec/cm^2^/sr/plasmid copy was observed confirming the transfectability of LLC tumours by this method (figure 2b).

Tumour growth was monitored by alternate day measurements in two dimensions using a Verniers callipers. Tumour volume was calculated according to the formula V = (ab^2^)∏/6. At each time point, a two-sampled t-test was used to compare mean tumour volume within each treatment group. Microsoft Excel (Microsoft) was used to manage and analyze data. Statistical significance was defined at the standard 5% level. Figure 2c shows tumour growth curves for the various groups (n = 6). While the Nk4 treated group showed a reduction in tumour size when compared with the control groups, the difference was not statistically significant. Three sample mice (external to measurement groups) from each group were culled at day 21 for analysis of lung metastases. Immediately post culling by cervical dislocation, mouse lungs were excised and fixed in Bouin's solution (Sigma, Dublin, Ireland) overnight to visualise metastatic foci macroscopically (figure 2d). No statistical significance was observed between groups in average numbers of macroscopic lung metastases. As some untreated mice had fewer but larger metastases than the treated group, the metastases volume was determined by measurement of the volume of the nodules (using a Verniers callipers and calculated as before). The Nk4 treated group had significantly reduced average metastatic burden when compared with the untreated control group in this context (p = 0.03) (figure 2e).

While both plasmid and adenoviral vectors have been utilised for Nk4 gene therapy of cancer [9-11], the short lived expression in tumours associated with these vectors may reduce therapeutic efficacy. AAV shows promise for anti-angiogenic gene therapy as it has been demonstrated that this vector can maintain gene expression for over 1 year [12-14] and elicits no cell-mediated immune response. To assess if prolonged and increased levels of expression at later time points would improve therapeutic responses, AAV2 mediated delivery of the Nk4 cassette was examined. The recombinant plasmids pAAV-Nk4 and pAAV-BB, were constructed as described in figure 1. AAVCMVLuc was generated by cloning of the Nco1, Xba1 fragment of pGL3 (Promega) containing the firefly *luciferase *gene, by blunt end ligation in the EcoRI, Xba1 region downstream of the CMV promoter of AAV-MCS cloning vector (Stratagene). AAV2 vector particles were prepared using the Stratagene AAV Helper Free System (Techno-Path, Limerick, Ireland), and concentrated using the Virakit system (Virapur, California, USA). Cells transduced with AAV-LacZ (Stratagene) particles were assessed for β-Galactosidase activity microscopically to determine the titre of the particle stocks, in parallel with AAV-Nk4 and AAV-BB. Nk4 expression was validated by reverse transcription PCR (RT-PCR) (Omniscript RT kit (Qiagen)) using primers forward CCTCTCTGATGACATGAAGAAG and reverse TGTCACAAAAGCTCTCCCC.

**Figure 3:**
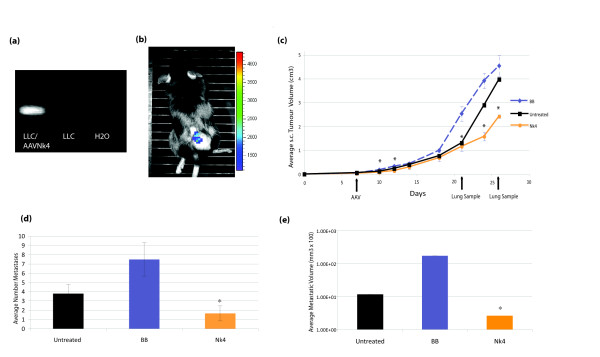
**Subcutaneous and metastatic LLC growth following AAV-mediated Nk4 Gene Therapy**. ***(a) AAVNk4 expression in vitro ***LLC cells were transduced with AAVNk4 particles *in vitro*. cDNA was prepared from total RNA extracted at 48 h, and subjected to Nk4 specific RT-PCR as before. Lane 1 = RNA from LLC transduced with AAVNk4 particles, lane 2 = RNA from untransduced LLC cells, lane 3 = H_2_O template control. **(b) *AAV transduction of LLC tumours ****In vivo *luminescence in LLC tumour following i.t. adminstration of AAV-CMV *Luc *particles. Image from IVIS Imaging System showing luciferase expression on day 9 post i.t. injection of AAV-*Luc *particles (2.91 × 10^-3 ^p/sec/cm^2^/sr/particle). **(c) *In vivo treatment of growing LLC tumours with AAV-Nk4 ***Established LLC tumours were i.t. administered AAV-Nk4 or AAV-BB (control) or no particles (PBS) and growth monitored (n = 6). Tumour growth in the AAV-Nk4 group was significantly reduced (*p < 0.05) when compared with the AAV-BB injected control group, and while the untreated group growth was also higher than the Nk4 group, it proved to be statistically insignificant. **(d) *Number of lung metastases following AAV-Nk4 therapy ***Visual analysis and comparison of surface metastatic nodules at days 21 and 26 showed that the Nk4 treated group had fewer nodules compared with the BB and untreated control group. A statistically significant difference could be seen between the combined day 21 and day 26 data from the Nk4 treated group and the BB control group (p = 0.015) (n = 3/group/timepoint). ***(e) Volume of lung metastases following AAV-Nk4 therapy ***Metastatic nodules were measured at days 21 and 26 and the average volume calculated. Both control groups had a larger metastatic burden than the Nk4 treated group with a significant difference between the Nk4 and untreated group (p = 0.021) and the BB group (p = 0.012).

Subcutaneous LLC tumours were induced in C57 mice and at an average volume of 0.1 cm^3^, i.t. administered 10^7 ^particles/40 μl AAV-Nk4, AAV-BB or PBS (n = 9). Tumour volumes were measured at regular intervals and 3 mice of each group were culled at 2 time points during the trial for analysis of lung metastases. The effects of AAV particles on tumour growth are detailed in figure 3. The s.c. tumour growth curve illustrates a marked decrease in growth in the AAV-Nk4 treated group in comparison with the untreated group and the AAV-BB control group. Unexpectedly, the backbone DNA containing AAV appeared to increase s.c. tumour growth, although not significantly. Significant difference was observed on days 10, 12, 21, 24 and 26 between the AAV-Nk4 and AAV-BB group (p < 0.05). Significant difference between the Nk4 treated and untreated group was approached towards the latter stages of the experiment but the trial had to be discontinued due to the tumour burden in the control groups in order to comply with ethical guidelines.

Pulmonary metastatic burden was assessed by visual counts at day 21 and 26. Mice in the control groups (BB and Untreated) showed more metastatic burden on both time points than the AAV-Nk4 treated group, and the BB group displayed increased (but statistically insignificant) metastatic burden over the untreated group (data not shown). Combined Day 26 and Day 21 measurements are shown in figures 3d &3e. Statistically significant differences in average numbers of lung metastases were seen between the Nk4 and BB group (p = 0.015) (figure 3d). When volumes of metastatic nodules were measured, the overall metastatic burden was significantly lower in the AAV-Nk4 treated group compared with both control groups; BB control group (p = 0.012), untreated group (p = 0.021) (figure 3e). It is unknown why AAV-BB increased tumour growth and metastases, and this was not observed in the plasmid experiments, suggesting that it is not as a result of DNA sequence, at least at the level of plasmid-mediated expression. Nevertheless, it cannot be ruled out that elements of the AAV vector were counteracting the therapeutic efficacy of Nk4.

It has previously been reported that by day 6 post tumour inoculation, 100% LLC mice have already developed metastatic disease [15]. In our trials, the earliest possible day of AAV injection into the tumours (minimum injectable size 0.1 cm^3^) was day 7. Others have addressed the limitation associated with AAV delayed expression by the use of self-complementary AAV [16]. It is plausible that increased therapeutic efficacy might be observed by achieving gene expression earlier in tumour growth and spread. The AAV2/2 serotype used in our studies has only a 30% reported efficiency of transducing LLC *in vitro *[17]. We observed an even lower efficiency (data not shown). Administration of a higher dose of AAV particles may increase effects on tumour growth and metastasis. This notwithstanding, AAV achieved dramatically higher expression levels per gene copy than plasmid (10^-3 ^p/sec/cm^2^/sr/AAVparticle vs 10^-8 ^p/sec/cm^2^/sr/plasmid copy). The significant differences in effects on tumours between the Nk4 containing and Nk4-free controls, coupled with demonstration of *in vivo *reporter gene expression in LLC tumours, as well as *in vitro *Nk4 expression data, indicate that Nk4 sequences were responsible for the observed effects on tumour growth.

Duration of gene expression is an important factor to be addressed in such gene therapies. It has previously been reported that slow release of NK4 plasmid DNA from cationised gelatin increases efficacy of Nk4 plasmid therapy [18]. While we did not investigate whether the superior responses observed with AAV over plasmid were as a result of increased duration or level of AAV expression, it is possible that a combination of the two systems described here may result in both immediate and long-term therapeutic expression enabled by plasmid initially, then to be superseded upon AAV activation.

The nature of our LLC model meant that it was not possible to generate survival curves based on death due to metastatic disease, as trials had to be stopped at or prior to 26 days post tumour inoculation due to primary tumour size. Plasmid experiments were ceased at day 21, due to early ulceration of tumours at subsequent times in plasmid administered groups, possibly related to toxicity of lipofectamine. No such ulceration was observed in AAV administered tumours up to day 26. A tumour model permitting longer-term study of this therapy would yield further information. Given the distance from clinical reality of fast-growing murine tumour models, anti-metastatic therapy as described here may yet prove a powerful therapeutic strategy in humans, especially if applied earlier in tumour progression.

## Competing interests

The authors declare that they have no competing interests.

## Authors' contributions

AB performed the *in vitro *and *in vivo *experiments, and contributed to drafting the manuscript. SAC and SR aided in generation of AAV vector particles and *in vivo *trials. JPvP designed and aided in cloning of AAV plasmids. MM constructed AAVCMVluc. GCO'S, DMO'H and MT were the coordinators of the project. MT designed the studies and drafted the manuscript. All authors read and approved the final manuscript.
